# Decoding the Therapeutic Implications of the ERα Stability and Subcellular Distribution in Breast Cancer

**DOI:** 10.3389/fendo.2022.867448

**Published:** 2022-04-13

**Authors:** Angeles C. Tecalco-Cruz, Marina Macías-Silva, Josué Orlando Ramírez-Jarquín, Uri Nimrod Ramírez-Jarquín

**Affiliations:** ^1^ Posgrado en Ciencias Genómicas, Universidad Autónoma de la Ciudad de México (UACM), Mexico City, Mexico; ^2^ Instituto de Fisiología Celular, Universidad Nacional Autónoma de México (UNAM), Mexico City, Mexico; ^3^ Neural Signal Transduction, Max Planck Florida Institute for Neuroscience, Jupiter, FL, United States; ^4^ Instituto Nacional de Cardiología “Ignacio Chávez”, Mexico City, Mexico

**Keywords:** estrogen receptor alpha, breast cancer, ERα stability, ERα nucleo-cytoplasmic transport, endocrine resistance, therapeutic approaches

## Abstract

Approximately 70% of all breast cancer cases are estrogen receptor-alpha positive (ERα+) and any ERα signaling pathways deregulation is critical for the progression of malignant mammary neoplasia. ERα acts as a transcription factor that promotes the expression of estrogen target genes associated with pro-tumor activity in breast cancer cells. Furthermore, ERα is also part of extranuclear signaling pathways related to endocrine resistance. The regulation of ERα subcellular distribution and protein stability is critical to regulate its functions and, consequently, influence the response to endocrine therapies and progression of this pathology. This minireview highlights studies that have deciphered the molecular mechanisms implicated in controlling ERα stability and nucleo-cytoplasmic transport. These mechanisms offer information about novel biomarkers, therapeutic targets, and promising strategies for breast cancer treatment.

## Introduction

Breast cancer is a collection of malignant mammary neoplasms that cause death in women worldwide (1–4). Breast cancer is classified in the subtypes luminal A, luminal B, HER2-overexpression, and basal-like (triple-negative) subtype, based on the detection mainly of ERα, PR, and HER2 expression by immunohistochemistry analysis (5). ERα (ERα+ breast cancer) is expressed in the luminal A/B and represents more than 70% of all cases of breast cancer (6, 7). Therefore, ERα detection is central in breast cancer tumors and is a target of some endocrine therapies, such as selective estrogen receptor downregulators (SERD) and selective estrogen receptor modulators (SERMs). Aromatase inhibitors (AI) are also used in endocrine therapy; however, they control the production of estrogens. A problem with these therapies is that patients develop *de novo* or *acquired* resistance (8).

ERα is a 66 kDa protein, a member of the nuclear receptor subfamily that is encoded by the *ESR1* gene, displaying conserved domains such as two activation function domains (AF-1 and AF-2), one DNA-binding domain (DBD), and one ligand-binding domain (LBD) (9–12). Furthermore, ERα contains nuclear localization signals (NLS) in the hinge region and nuclear export signals (NES) in DBD and LBD (13–15). The structure and function of ERα are modulated by different posttranslational modifications, such as the phosphorylation of the AF-1 domain induced by E2 (estradiol) but also induced *via* growth factor signaling (16–19). This minireview is focused mainly on the molecular mechanisms that modulate the nucleo-cytoplasmic transport and stability of ERα in breast cancer.

## ERα Signaling and Its Nucleo-Cytoplasmic Dynamics in Breast Cancer

**Figure 1:**
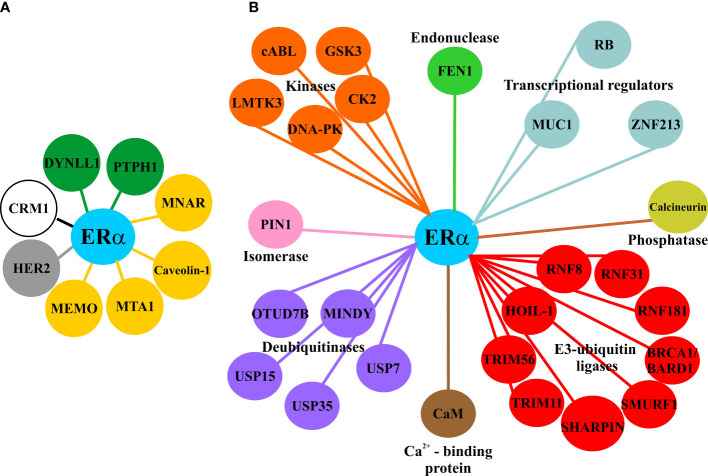
Proteins related to subcellular transport of ERα and its stability in breast cancer cells. **(A)** Principal proteins involved in the nuclear accumulation of ERα in the nucleus (green), in the extranuclear localization of ERα (yellow), required for the nuclear export (white), and correlated with the nuclear translocation of ERα (gray). **(B)** Interactome of proteins that increase the stability of ERα in breast cancer cells.

ERα is localized in both the cytoplasm and the nucleus of breast cancer cells. The ERα canonical signaling pathway consists of the binding of E2 to the receptor LBD, triggering its homodimerization, enrichment into the nucleus, binding to estrogen-responsive element (ERE) in enhancers or promoters of E2-responsive genes, and recruitment of coregulators *via* the AF1/2 domains to induce gene expression (20, 21). Pioneer FTs open up local chromatin, allowing ERα to interact with ERE and recruit coregulators to modulate chromatin structure and gene expression (22). Coregulators are recruited by the AF-1 and AF-2 domains in an E2-independent and -dependent manner, and they are important for the interactions between ERα-dependent enhancers and promoters to synergistically regulate transcription in breast cancer cells (23–25). ERα also acts as a coregulator for diverse TF such as AP-1/c-Jun, ATF-2, NF-kappaB, p53, SP-1, and STAT1, modulating the expression of several genes, including late E2-target genes (16, 26–29). ERα can act as a coregulator when it is phosphorylated in response to growth factors, generating a crosstalk with other signaling pathways (30–35). It has been reported that the levels of DLC1 (dynein light chain 1) are increased in breast cancer and that DYNLL1, also named DLC1, promotes ERα nuclear accumulation and its activity in response to E2 (36) (**Figure 1**). In addition, ERα is membrane-associated *via* its palmitoylation, having the ability to respond to E2 at 3-15 min, generating secondary messengers such as Ca^2+^, cAMP, and nitric oxide. ERα also interacts with transmembrane receptors, such as RTK (receptor tyrosine kinases), GABAB, and mGluR (37–41).

The nuclear export of ERα is mediated by non-canonical NES in the DBD and LBD, which are recognized by CRM-1 exportin, being an E2-dependent process in breast cancer cells (13, 15). The ERα Y537F mutant is unable to associate with CRM-1, resulting in its nuclear accumulation (42). The Y537 is the phosphorylated site by Src, and the treatment with a Src inhibitor (SU6656) or the expression of a dominant-negative Src protein decrease E2-induced ERα phosphorylation and nuclear export (15, 42, 43). In addition, the use of the CRM-1 inhibitor, LMB, decreases ERα transactivation, suggesting that a nucleo-cytoplasmic dynamic is required for ERα nuclear activity (44).

Moreover, E2 induces AKT-dependent phosphorylation of FKHR, promoting the nucleo-cytoplasmic transport of the ERα/FKHR complex (15, 43). In contrast, ATBF1 is another transcription factor enriched in the nucleus of MCF-7 cells in response to E2 hormone and in an ERα-dependent manner, whereas ATB1 is localized in the cytoplasm in those breast cancer cell lines that do not express ERα (45). These data suggest that the subcellular dynamics of some transcription factors may be dependent on ERα status.

**Figure 2:**
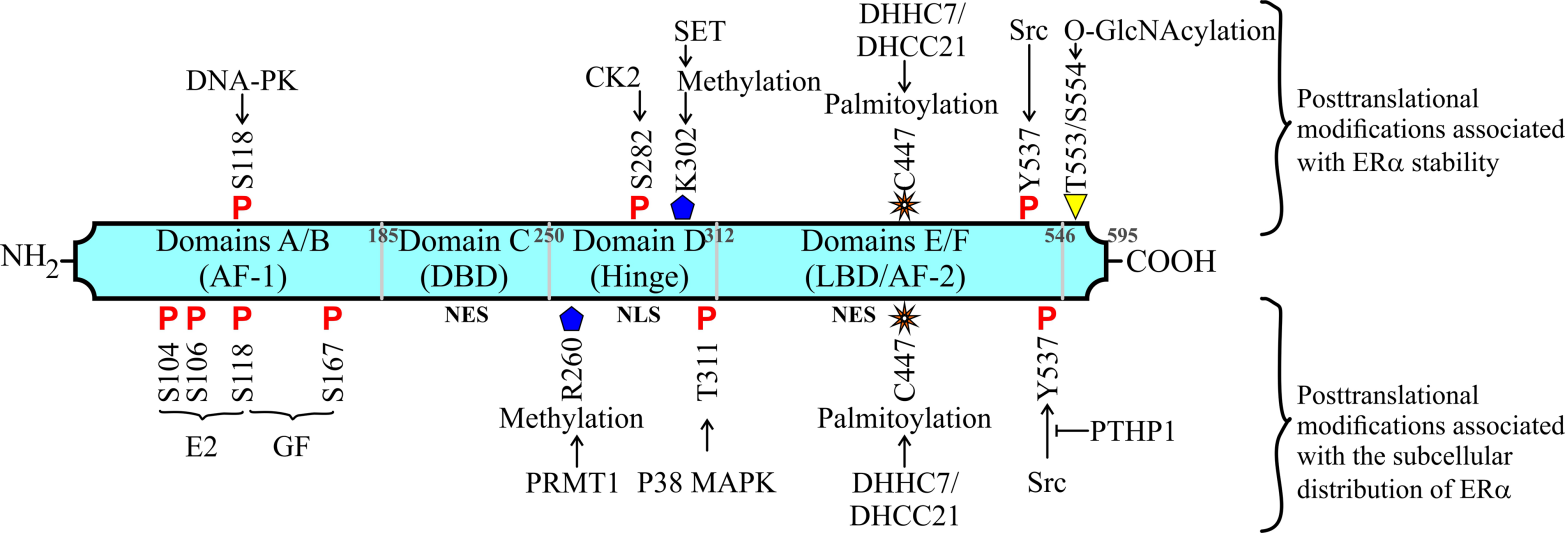
Other posttranslational modifications related to subcellular transport and stability of ERα in breast cancer. Structure of ERα protein and its functional domains. Up: Modifications related to ERα stability. Down: Modifications involved in the subcellular transport in breast cancer cells.

In addition, the extranuclear localization of ERα is facilitated by its interaction with proteins such as MEMO (ErbB2-driven cell motility), MNAR (modulation of non-genomic actions of the estrogen receptor), and MTA1 (metastasis-associated 1). MEMO increases Y537 phosphorylation in the ERα and enhances cell proliferation and migration (46) (**Figure 1**). MNAR and truncated MTA1 sequester ERα and increase its activities out of the nucleus (47, 48). In contrast, the accumulation of ERα in the nucleus is promoted by PTPH1 (protein-tyrosine phosphatase H1) that reverts Src-dependent Y537 phosphorylation, and by the phosphorylation of T311 by p38 MAPK (49–51). Phosphorylated ERα at T311 has been found in human breast tumors (50), and the Y537S, Y537C, and Y537N mutations have been detected in metastatic mammary tumors that are resistant to endocrine therapies (52, 53) (**Figure 2**). ERα can interact with a signalosome complex that includes c-Src, PI3K, caveolin-1, straitin, and MNAR (54–56).Caveolin-1, a protein enriched mainly in caveolae, interacts with ERα, leading to the trafficking of ERα to caveolae to promote its localization on plasma membrane and the activation of non-genomic pathways (56, 57).

### ERα Distribution and Its Relationship With Therapeutic Approaches for Breast Cancer

Some studies suggest that ERα nuclear distribution is associated with the sensitivity of breast cancer cells to endocrine therapy, whereas extranuclear localization is related to endocrine resistance. For example, Selinexor is a CRM-1 inhibitor that combined with tamoxifen can restore the sensitivity of cells to tamoxifen (58). In addition, when PTPH dephosphorylates Y537, ERα is enriched in the nucleus, and breast cancer cells are sensitive to tamoxifen and fulvestrant (49). Another example is the use of Src inhibitors; among them, dasatinib, in combination with tamoxifen, restores the nuclear distribution of ERα and the sensitivity to endocrine therapy of tamoxifen-resistant cells (59, 60). Moreover, MCF-7 breast cancer cells that overexpress HER2 display an ERα translocation nucleo-cytoplasm and are resistant to tamoxifen (61–63). Nuclear redistribution of ERα and re-sensitivity to endocrine therapy are recovered using the HER2 inhibitor AG825 and anti-HER2 monoclonal antibody (61, 62).

Additionally, the methylation of ERα at R260 *via* PRMT1 (arginine methyltransferase) allows the formation of methyl-ERα/Src/PI3K complex in response to estrogens. The interactions ERα/Src/PI3K is enhanced in aggressive mammary malignant tumors, promoting non-genomic signaling related to resistance to tamoxifen and poor survival. Hence, methyl-ERα/Src/PI3K complex has been proposed as a hallmark of aggressiveness and resistance to tamoxifen. Consequently, the disruption of functional interaction between ERα and PI3K, using the combination of Src or PI3K inhibitors plus tamoxifen or fulvestrant, has been proposed as a strategy in the treatment of ERα+ breast cancer (64, 65).

## ERα Is Modulated *via* Its Mono-Ubiquitination and Polyubiquitination

The polyubiquitination of ERα at K302/K303 is induced by E2 and fulvestrant and is associated with its degradation *via* the UPS (66). However, the turnover of ERα induced by E2 is also important for its activity, since it has been reported that the inhibition of ERα degradation reduces the recruitment of RNA polymerase II to ERE, and the intranuclear dynamic of ERα is affected by transcriptional or proteasome inhibitors (67, 68). Moreover, ERα coactivators such as E6AP, RNF8, and SKP2 also function as E3-ubiquitin ligases, suggesting an intriguing interplay between ERα transcriptional activity and its polyubiquitination/degradation (29, 67, 69–72).

ERα monoubiquitination at K302/K303 residues modulates cell proliferation induced by E2 (73–76). These monoubiquitinations catalyzed by BRCA-1/BARD1 confers receptor stability under basal conditions (66, 73, 76). It has also been reported that E2 inhibits ERα monoubiquitination (77). In addition, UBD or ubiquitin-binding domains have been identified in the LBD of ERα *(*L429 and A430 residues), allowing the association of this receptor with ubiquitinated proteins. ERα monoubiquitination and its activity are affected when UBD is mutated (78, 79).

### ERα Stability in Breast Cancer

Several studies have identified proteins that interact with ERα and inhibit its polyubiquitination and degradation (**Figure 1**). The primary functions of the ERα-polyubiquitination inhibitor proteins (EPIP) vary from being transcriptional coregulators, kinases, E3-ubiquitin ligases, or deubiquitinases (**Table 1**). Most of them are upregulated in breast cancer tissue, promoting ERα stability and breast cancer progression. Thus, proteins promoting ERα stability facilitates higher levels of this receptor, and its actions are associated with the expression of its target genes, cell proliferation, and endocrine resistance (71, 92, 94). One example of those proteins is the endonuclease FEN1, which is increased in tamoxifen-treated breast cancer patients, promoting the transcriptional activity of ERα. Moreover, FEN1 inhibits ERα degradation and maintains its stability to increase the expression of its target genes and cell proliferation. Inhibition of FEN1 decreases ERα activity and proliferation in breast cancer cells resistant to tamoxifen, suggesting the therapeutic potential of FEN1 as a target molecule in endocrine therapy resistance (101). Another example of EPIP is calcineurin, a Ca^2+^-dependent protein phosphatase, which dephosphorylates the Ser294 in ERα to inhibit its degradation *via* the UPS. Moreover, calcineurin facilitates the ERα phosphorylation at Ser118 by mTOR to increase its activation. A higher expression of calcineurin is associated with a poor prognosis in patients receiving endocrine therapy, suggesting that it is a key target for breast cancer treatment (102).

**Table 1:** Principal proteins involved in the ERα stability and subcellular transport in breast cancer cells.

Proteins associated with ERα stability			
Protein	Name	Function	Reference(s)
cABL	Abelson tyrosine-protein kinase	Kinase	(80)
GSK3	Glycogen Synthase Kinase 3	Kinase	(81)
LMTK3	Lemur Tyrosine Kinase 3	Kinase	(82)
DNA-PK	DNA-dependent protein kinase	Kinase	(83)
CK2	Casein kinase 2	Kinase	(84)
PIN1	Peptidyl-propyl cis-trans isomerase NIMA-interacting 1	Isomerase	(85)
MINDY	Motif interacting with ubiquitin-containing novel DUB family	Deubiquitinase	(86)
OTUD7B	OTU Deubiquitinase 7B	Deubiquitinase	(87)
USP7	Ubiquitin-specific protease 7	Deubiquitinase	(88)
USP15	Ubiquitin-specific protease 15	Deubiquitinase	(89)
USP35	Ubiquitin-specific protease 35	Deubiquitinase	(90)
HOIL-1	Haem-oxidized IRP2 Ubiquitin Ligase-1	E3-ubiquitin ligase	(91)
RNF8	RING finger protein 8	E3-ubiquitin ligase	(71)
RNF31	RING finger protein 31	E3-ubiquitin ligase	(92)
RNF181	RING finger protein 181	E3-ubiquitin ligase	(93)
SHARPIN	Shack-associated RH domain-interacting protein	E3-ubiquitin ligase	(94)
SMURF1	SMAD ubiquitination regulatory factor	E3-ubiquitin ligase	(95)
TRIM11	Tripartite Motif Containing 11	E3-ubiquitin ligase	(96)
TRIM56	Tripartite Motif Containing 56	E3-ubiquitin ligase	(97)
BRCA-1/BARD1	Breast cancer type 1/BRCA1 associated RING domain 1	E3-ubiquitin ligase	(73, 76)
RB	Retinoblastoma	Tumor suppressor Transcriptional regulator	(98)
MUC1	Mucin 1	Transcriptional regulator	(99)
ZNF213	Zinc finger protein	Transcriptional regulator	(100)
FEN1	Flap Structure-Specific Endonuclease 1	Endonuclease	(101)
Calcineurin	Calcium and Calmodulin dependent serine/threonine protein phosphatase 2B.	Phosphatase	(102)
CaM	Calmodulin	Multifuntional Ca^2+^-binding protein	(103, 104)
**Proteins associated with the subcellular distribution of ERα**			
CRM1	Chromosomal Maintenance 1	Exportin	(105)
DYNLL1	Dynein light chain 1	Motility	(36)
MEMO	Mediator of ERBB2-driven cell motility	Motility	(46)
MNAR	Modulator of non-genomic activity of estrogen receptor	Scaffold	(47)
MTA1	Metastasis-associated protein MTA1	Transcription regulator	(48)
PTPH1	Protein Tyrosine Phosphatase H1	Phosphatase	(49)
HER2	Human epidermal growth factor receptor 2	Transmembrane receptor	(61, 62)
Cav1	Caveolin-1	Protein of caveolae	(57)

Some EPIPs are E3-ubiquitin ligases that appear to play a complex role in stabilizing the ERα *via* different mechanisms. For example, most TRIMs (tripartite motif-containing) act as E3-ligases. In breast cancer, TRIM11 and TRIM56 confer ERα stability (96, 97), whereas TRIM8 increases ERα degradation in the cytoplasm (106). Furthermore, TRIM11, RNF8, RNF31, and SHARPIN catalyze the ERα monoubiquitination and inhibit its degradation (71, 92, 94, 96). Smurf1, TRIM56, and HOIL-1 block ERα degradation by inhibiting K48-specific polyubiquitination (91, 95, 97), whereas RNF181 induces K63-linked ubiquitination, which stabilizes ERα in BC cells (93).

Interestingly, some kinases affect the activity and stability of ERα receptor. For example, the LMTK3, GSK3 and cABL kinases interact with and phosphorylate ERα, avoiding its degradation (80–82). DNA-PK (DNA-dependent protein kinase) phosphorylates ERα at Ser-118 to stabilize it, promoting its transcriptional activity, and the proliferation of breast cancer cells (83). Furthermore, the S282 residue of ERα can be phosphorylated by CK2, resulting in the stability of this receptor in breast cancer cells (84).

Proteins with deubiquitinase activity are also central to regulate ERα stability in breast cancer, such as USP7, USP15, USP35, OTUD7B, and MINDY. For example, MINDY has a positive correlation with ERα levels, and promotes poor prognosis in breast cancer by stabilizing the ERα *via* the inhibition of its K48-polyubiquitination (87).

Furthermore, the calmodulin (CaM) protein modulates ERα transactivation in a Ca2+-dependent manner (107, 108). The residues Pro-295 to Ser-317 localized between hinge and LBD of ERα are central for binding of CaM. Mutations in these sites decrease the ERα interaction with CaM and the E2-dependent gene transcription (108–110). Studies using a synthetic peptide containing these major determinants (ERα17p: P295-T311) compared to control peptides with Lys-302 and Lys-303 mutated to alanines or glycines (ERα17pAA or ERα17pGG) evidenced that this sequence has an auto-inhibitory activity, which may be relieved by CaM binding (103, 104, 109, 110). Hence, this ERα motif seems to be essential to interact with proteins implicated in its regulation. Interestingly, CaM interacts with ERα and protects it from proteolysis by inhibiting the E6AP-dependent degradation of this receptor (111, 112).

Posttranslational modifications, such as methylation (by SET7 at K302) and palmitoylation (by DHHC7 and DHHC21 at C447) also contribute to ERα stability, inhibiting its degradation (37, 113, 114). In addition, O-GlcNAcylation at T553/S554 residues in ERα mediated by GREB inhibits ZNF598 ubiquitin ligase-dependent degradation, leading to ERα stability (115). In addition, other stimuli, such as the aluminum salts present in antiperspirants, have been associated with ERα stability and accumulation in the nucleus, with an increase in gene expression (116). However, ERα stability is also conferred *via* indirect mechanisms. For example, PEBP4 (phosphatidyl-ethanolamine-binding protein 4) decreases ERα degradation induced by its Src-dependent phosphorylation, since PEBP4 inhibits the association between Src and ERα (117). The Y537 residue in ERα is phosphorylated by Src kinase to recruit the E6AP protein, which is an E3-Ub ligase that polyubiquitinates ERα for its degradation in breast cancer cells. The interaction of PIN1 with ERα inhibits its phosphorylation (at Y537) and its interaction with E6AP, conferring stability (70, 85, 117).

The proteolysis of ERα can be affected by ERα protein accumulation (118–120), which leads to non-classical mechanisms called *concentration-inducible* ERα *function*, where ERα is active in a manner stimuli-independent (E2 signal, or growth factor signals), promoting changes in the expression of its target genes, resulting in new E2-induced genes (121, 122). These data suggest that alterations in the interplay of proteolysis and stability of ERα may have crucial implications in malignant mammary tumors.

Although the higher levels of ERα by increasing its stability are associated with cancer progression and endocrine resistance, the reduction of ERα levels by an increase in its degradation is also related to endocrine resistance, considering that ERα is the target for SERMs and SERDs. Hence, CUEDC2 induces ERα degradation *via* the UPS, and some malignant mammary tumors with resistance to tamoxifen show high levels of CUEDC2 protein with low levels of ERα (123, 124). In contrast, RB is a protein that stabilizes ERα and protects it from its degradation. Increased ERα degradation through the UPS has been reported in RB-knockdown breast cancer cell lines (98), whereas ERα– mammary tumors display alterations in the expression and function of RB (125, 126).

### ERα Stability and Its Relationship With Therapeutic Approaches

Fulvestrant, a SERD clinically used as first-line endocrine therapy to inhibit tumor growth, promotes ERα polyubiquitination and degradation. Other SERDs are being investigated to improve their effects, availability, and administration routes (127–133). Intriguingly, when the expression of large tumor suppressor kinases 1 and 2 (LATS1 and 2) is reduced, the sensitivity to fulvestrant of breast cancer cells is decreased. LATS1/2 (two mediators of the Hippo pathway) are associated with the induction of ERα degradation. High levels of LATS1/2 are detected in patients with breast cancer ERα– and short relapse-free survival (134).

ERα mutations, such as Y537S/N/C, D538G, E380Q, or S463P have been associated with endocrine resistance. In particular, the mutations Y537S, Y537N, Y537C, D538G, and E380Q localized in the LBD of ERα cause an E2-independent activity of ERα (135, 136). These mutations have been detected mainly in metastatic breast cancer (137) and affect gene expression (138) and ERα-dependent cistrome (139). Mutations in the Y537 residue (Y537S, Y537C, and Y537N) can affect the degradation of this receptor, which is associated with metastasis and resistance to endocrine therapy in patients (52, 53, 70, 140). After cells acquire endocrine resistance, Y537C and Y537S are detected, which may be due to long-term E2 deprivation (141).

Mutations in the Y537 residue do not affect fulvestrant and AZD9496 treatments, suggesting the use of SERD to treat endocrine resistance. However, an interesting study showed that when the E2-induced polyubiquitination of ERα is decreased, the ERα stability is increased only in invasive lobular breast carcinoma but not in invasive ductal carcinoma. Fulvestrant was effective in both breast cancer subtypes; however, the SERD AZD9496 does not have the same effect in the reduction of ERα stability in invasive lobular breast carcinoma, suggesting that ERα stability and its functional implications are regulated differentially by SERD therapies in both histological subtypes of breast cancer (142).

In addition to SERDs, other modulators of ERα that diminish its stability are being studied. For example, MHO7 (6-epi-ophiobolin G) is a compound that inhibits the synthesis of ERα mRNA and increases the degradation of this receptor *via* the UPS, postulating it as a drug candidate to promote ERα downregulation and block breast cancer progression (143).

## Discussion

Most cases of breast cancer are ERα+, where this receptor displays pro-tumoral activity, and the molecular mechanisms that regulate its activity are crucial. Some patients with breast cancer have or develop resistance to SERMs and AI, whereas the treatment with SERDs as fulvestrant is not affected by mutations in ERα related to endocrine therapy. The anti-tumor effect of SERDs is based on ERα degradation *via* the UPS. Interestingly, E2 induces ERα degradation through UPS, both in the cytoplasm and nucleus, whereas fulvestrant induces the degradation of this receptor in the nuclear matrix. Additionally, ERα protein can be downregulated by E2-dependent lysosomal degradation (144), dynamin II-dependent autophagy (145), and *via* its association with caveolin 1/2 (146), and the clathrin-heavy chain (CHC) endocytic protein (147).

In recent years, many investigations on ERα stability and its nuclear export in breast cancer suggest that these events affect the nuclear and extranuclear activity of this receptor and the cell response to endocrine therapies. For example, Src-dependent phosphorylation at Y537 is required for nuclear export and E6AP-dependent degradation in breast cancer cells, suggesting that ERα subcellular distribution may be associated with its stability (42, 70, 140, 148). Posttranslational modifications of ERα, such as phosphorylation and poly-/mono-ubiquitination, appear to be central for the modulation of its stability, transport, and localization, and some may compete by the same site to modulate ERα stability and activity; for example, K303 is acetylated, mono- and poly-ubiquitinated in breast cancer cells (66, 73, 76, 113, 149), and some mutations at K303 exist in premalignant breast lesions (150, 151). Moreover, many proteins participate to protect ERα from degradation and affect its subcellular distribution in breast cancer, denoting a complex interplay among these elements, and some of them may be potential therapeutic targets. Furthermore, all data indicate that the response to endocrine therapy requires a dynamic in ERα stability/degradation and its subcellular transport.

ERα proteolysis is key to the design of new therapeutic strategies to treat breast cancer, such as PROTACs (proteolysis targeting chimeric) technology, which are modulators of ERα and its mutants (136, 152). PROTACs contain a module for binding to the target protein and another module for the recognition of E3 ligase. Hence, PROTACs bind to their target protein to promote its ubiquitination and degradation, and different PROTACs have been developed to degrade ERα *via* the UPS in breast cancer cells, exhibiting antitumor activity. PROTACs are being evaluated in patients with metastatic breast cancer and may become promising therapies (153). It is important to consider the implications of ERα stability in malignant mammary neoplasia to avoid some resistance to SERD or PROTAC treatments.

In conclusion, more studies focusing on ERα stability and nuclear export in breast cancer are required. However, several investigations have emerged to date, indicating that therapeutic strategies based on controlling ERα abundance and distribution in breast cancer may improve the status of patients with endocrine resistance.

## Author Contributions

All authors listed have made a substantial, direct, and intellectual contribution to the work and approved it for publication.

## Funding

Our work is partially supported by institutional budget from UACM and IFC at UNAM.

## Conflict of Interest

The authors declare that the research was conducted in the absence of any commercial or financial relationships that could be construed as a potential conflict of interest.

## Publisher’s Note

All claims expressed in this article are solely those of the authors and do not necessarily represent those of their affiliated organizations, or those of the publisher, the editors and the reviewers. Any product that may be evaluated in this article, or claim that may be made by its manufacturer, is not guaranteed or endorsed by the publisher.
